# CO_2_ – Intrinsic Product, Essential Substrate, and Regulatory Trigger of Microbial and Mammalian Production Processes

**DOI:** 10.3389/fbioe.2015.00108

**Published:** 2015-08-03

**Authors:** Bastian Blombach, Ralf Takors

**Affiliations:** ^1^Institute of Biochemical Engineering, University of Stuttgart, Stuttgart, Germany

**Keywords:** bicarbonate, carbon dioxide, production process, regulation, carboxylation, decarboxylation

## Abstract

Carbon dioxide formation mirrors the final carbon oxidation steps of aerobic metabolism in microbial and mammalian cells. As a consequence, ${\text{CO}}_{\text{2}}/{\text{HCO}}_{\text{3}}^{-}$ dissociation equilibria arise in fermenters by the growing culture. Anaplerotic reactions make use of the abundant ${\text{CO}}_{\text{2}}/{\text{HCO}}_{\text{3}}^{-}$ levels for refueling citric acid cycle demands and for enabling oxaloacetate-derived products. At the same time, CO_2_ is released manifold in metabolic reactions via decarboxylation activity. The levels of extracellular ${\text{CO}}_{\text{2}}/{\text{HCO}}_{\text{3}}^{-}$ depend on cellular activities and physical constraints such as hydrostatic pressures, aeration, and the efficiency of mixing in large-scale bioreactors. Besides, local ${\text{CO}}_{\text{2}}/{\text{HCO}}_{\text{3}}^{-}$ levels might also act as metabolic inhibitors or transcriptional effectors triggering regulatory events inside the cells. This review gives an overview about fundamental physicochemical properties of ${\text{CO}}_{\text{2}}/{\text{HCO}}_{\text{3}}^{-}$ in microbial and mammalian cultures effecting cellular physiology, production processes, metabolic activity, and transcriptional regulation.

## Introduction

One of the most decisive decisions which needs to be made when developing novel bioprocesses is whether the final process will run under anaerobic or aerobic conditions. While severely reduced investment costs speak in favor of anaerobic production, expected productivities and intracellular energy availabilities are drivers for aerobic approaches. Anaerobic metabolism yields at two net ATP produced in glycolysis per glucose while aerobic counterparts may achieve >12 ATPs. This net ATP yield even represents a conservative estimation considering true ATP per oxygen (P/O) ratios of 1:1.3 which are lower than theoretical maxima of 2–3. Consequently, aerobic processes are often the first choice if ATP-challenging product formation with maximum cell-specific formation rates is targeted.

Carbon dioxide (CO_2_) is the inevitable product of respiration processes and as such always present in aerobic bioprocesses. This holds also true for the production of commodities, fine chemicals, or therapeutic proteins using microbes or mammalian cells. While therapeutic proteins and fine chemicals are typically produced in bioreactor of 5–20 m^3^ scale, the production of commodities is usually performed in 50–500 m^3^ size – or even larger. As an intrinsic property, partial CO_2_ pressures of these scales differ significantly from those found in lab-scale. This phenomenon is the inherent consequence of high absolute pressures and poor mixing conditions in large-scale bioreactors (Takors, 2012).

CO_2_ and its hydrated counterpart ${\text{HCO}}_{3}^{-}$ may not only serve as substrate or product for carboxylating and decarboxylating reactions, the species may also alter physicochemical properties of proteins, acidify the internal pH, and regulate virulence and toxin production in pathogens (Follonier et al., 2013). Therefore, ${\text{CO}}_{\text{2}}/{\text{HCO}}_{\text{3}}^{-}$ can interact with cellular metabolism and can even create complex transcriptional responses. CO_2_ not only freely diffuses through the cellular membrane (Gutknecht et al., 1977), it may also accumulate in the same (Jones and Greenfield, 1982; Kuriyama et al., 1993; Bothun et al., 2004), thus increasing its permeability and fluidity which finally leads in the potentially lethal “anesthesia effect” (Isenschmid et al., 1995).

The fact that high pCO_2_ levels are used to sterilize food (Ballestra et al., 1996; Spilimbergo and Bertucco, 2003; Garcia-Gonzalez et al., 2007) anticipates that elevated pCO_2_ are not likely to improve the performance of microbial or mammalian production processes. Instead, as it will be shown, high pCO_2_ levels often coincide with the deterioration of the bioprocess performance. Consequently, thorough scale-up studies should preferably consider the analysis of pCO_2_ impacts to ensure an equally good performance in large-scale compared to lab-scale expectations. This is especially true for the establishment of novel bioprocesses which are the result of systems metabolic engineering studies performed in lab-scale.

This contribution aims at reviewing fundamental properties, sources, and impacts of CO_2_ for microbial and mammalian production processes. It yields at bringing together the major puzzle pieces of how ${\text{CO}}_{\text{2}}/{\text{HCO}}_{\text{3}}^{-}$ interacts with producer cells. It will show that a lot has already been done – but still not everything is fully understood. This holds especially true for regulation of cellular metabolism where ${\text{CO}}_{\text{2}}/{\text{HCO}}_{\text{3}}^{-}$ apparently serves as an underestimated trigger so far.

## Fundamentals – Physicochemical Properties and Mass Transfer

Carbon dioxide (CO_2_, molar weight: 44.01 g/mol) is a colorless, odorless gas of linear molecular shape with a melting point at −56.6°C. It is present in the Earth atmosphere as a trace compound currently showing levels of about 400 ppm with the tendency of steady increase (http://co2now.org/).

The water solubility can be described applying Henry’s law.

(1) $$H_{\text{CO2}}=\frac{c_{\text{CO2,L}}}{p_{\text{CO2}}}\left[\frac{\text{mmol}}{\text{L bar}}\right]$$

with *c*_CO2,L_ and *p*_CO2_ coding for the equilibrium values of the molar concentration of dissolved CO_2_ in the liquid L and the related partial CO_2_ pressure, respectively. For pure water at 25°C the Henry coefficient *H*_CO2_ = 34.5 mmol/barL is given (Stumm and Morgan, 1995). Using the Van’t Hoff correlation

(2) $$\frac{d\text{ln}H_{\text{CO2}}}{\mathrm{dT}}=\frac{\Delta H^{0}}{{\mathrm{RT}}^{2}}\Rightarrow H_{\text{CO2}}(T)=\text{ln}K-\frac{\text{Δ}H^{0}}{\mathrm{RT}}$$

the temperature dependency of the equilibrium constant (here: Henry-coefficient *H*_CO2_) can be estimated with the standard enthalpy change of the reaction Δ*H*^0^, the universal gas constant *R*, and the absolute temperature *T* as shown. Noteworthy, *K* codes for an integration constant that can be derived from reference data e.g., at 25°C. Using equation (2), *H*_CO2_(*T* = 20°C) = 40 mmol/barL and *H*_CO2_(*T* = 37°C) = 25 mmol/barL can be calculated. Decreasing Henry coefficients [as defined by (1)] mirror reducing gas solubility with rising temperature – a typical phenomenon for dissolved gasses at the given temperature range.

Besides temperature, CO_2_ solubility is also affected by electrolyte concentrations. Following the empirical Sechenov (1889) approach individual contributions of ion strength can be considered to estimate the resulting solubility of a gas in the salt-containing liquid (Noorman et al., 1992). However, the composition of fermentation media is often complex and changes steadily during the course of cultivation. Product and by-product formation, substrate consumption, and the addition of titrating agents are the reasons. Therefore, the most pragmatic approach is to measure CO_2_ solubility in real cultivation media. Own experimental observations show that real *H*_CO2_ values [according to (1)] are often increased, may be even doubled, compared to values for pure water (unpublished data).

**Figure 1:**
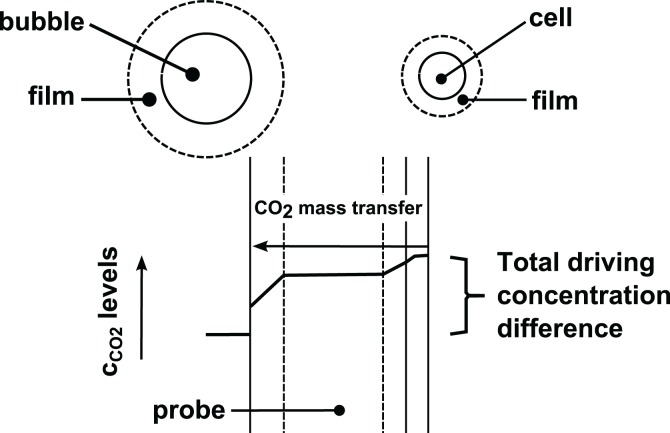
**Principles of CO_2_ mass transfer**. CO_2_ is released by the cells and transported through the surrounding liquid film via diffusion. After leaving this few μm-thick layer dissolved CO_2_ enters the well-mixed zone and is transported to the bubbles via convection. To reach the bubbles dissolved CO_2_ must pass the surrounding film by diffusion again. Probes for dissolved CO_2_ or O_2_ measurements are installed in the convective zone thus giving the related signal.

Applying typical operating conditions, microbial or mammalian cultivations release exhaust gas with volumetric CO_2_ fractions of 5–25%. For a conservative estimation, one can assume equilibrium conditions between gas and liquid with *H*_CO2_ values for pure water at 37°C. Then dissolved CO_2_ levels *c*_CO2,L_ are likely to range between 75 and 375 mg/L. For instance, Blombach et al. (2013) measured p_CO2_ levels of about 160 mbar (about 360 mg/L) at the end of an aerated (0.1 vvm) 1.5 bar pressured, stirred batch cultivation with 5 g_CDW_ *Corynebacterium glutamicum* per L. Increasing the aeration to 3 vvm reduced the p_CO2_ to 40 mbar (about 90 mg/L). Similar values were observed by Buchholz et al. (2014b). By contrast, maximum dissolved oxygen concentrations under atmospheric conditions will typically result at 7.5–8 mg/L (again depending on medium composition). Consequently, dissolved CO_2_ levels outcompete dissolved O_2_ levels by far. This finding may be even more pronounced if mass transport characteristics are considered (Figure 1).

Figure 1 shows that maximum *c*_CO2,L_ levels are found in the proximate microenvironment of the cells. By trend, probes for dissolved gas measurement observe lower levels. This is different compared to dissolved oxygen where cells face the lowest levels along the mass transfer path.

While dissolved carbon dioxide levels may achieve high inhibiting values during the fermentation course, starting conditions might be limiting instead. Assuming equilibrium between inlet aeration and the liquid 0.5 mg_CO2_/L is present. Noteworthy this low value is likely to persist if too high aeration (with low concentrated CO_2_) strips out new metabolically produced CO_2_. Consequently anaplerotic reactions may be limited by substrate ${\text{(HCO}}_{\text{3}}^{-}/{\text{CO}}_{\text{2}})$ supply (see Section “Metabolic Release and Incorporation”) finally resulting at reduced cell growth.

By analogy to oxygen transfer, the CO_2_ transfer rate *CTR* (mmol/Lh) can be described according to the following:

(3) $$CTR=k_{L}a_{\text{CO2}}\left(c_{\text{CO2,L}}^{*}-c_{\text{CO2,L}}\right)$$

with *k*_L_*a*_CO2_ coding for the CO_2_ mass transfer coefficient (1/h), $c_{\text{CO2,L}}^{*}$ for the dissolved CO_2_ concentration at equilibrium following Henry’s law (mmol/L) and *c*_CO2,L_ representing the measured concentration (mmol/L).

Measuring true *k*_L_*a*_CO2_ values in praxis is somewhat challenging. One approach is to assume *CTR* = *CER*, i.e., carbon dioxide emission rate *CER* equals the CO_2_ stripping rate *CTR*. By balancing flows of aeration and exhaust gas, related values should be accessible and *k*_L_*a*_CO2_ can be derived accordingly. Nevertheless, this approach reveals its drawback when mammalian cell cultures are balanced. Here, the exhaust gas signal is a superposition of biological activity and CO_2_ addition for titration. Alternatively, *k*_L_*a*_CO2_ could be estimated from *k*_L_*a*_O2_ according to the following:

(4) $$k_{\text{L}}a_{\text{CO2}}=k_{\text{L}}a_{\text{O2}}\sqrt{\frac{D_{\text{CO2}}}{D_{\text{O2}}}}$$

**Figure 2:**
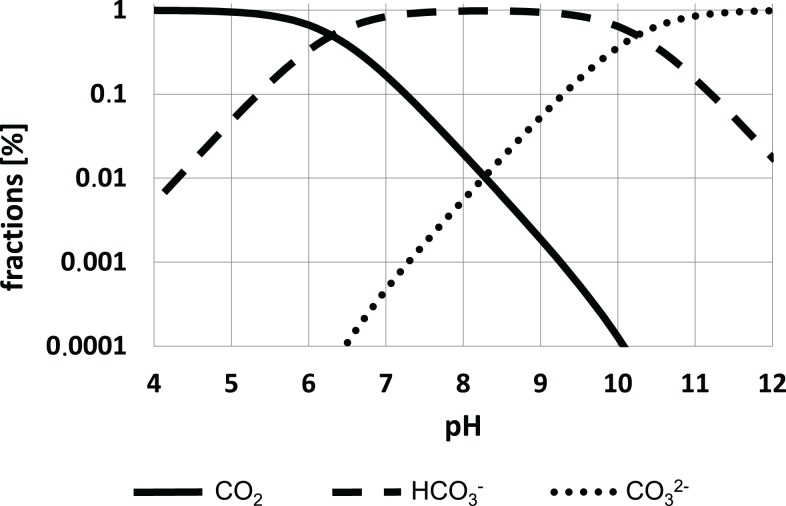
**Dissociation equilibria of CO_2_ species depending on the pH**. The equilibrium constants *K*_1_ = 10^−6.3^M and *K*_2_ = 10^−10.25^M were chosen. For details see text.

Equation (4) results from Higbie’s penetration theory Higbie (1935) and Danckwerts surface renewable model Danckwerts (1951). Apparently, the mass transfer coefficient for CO_2_ is proportionally linked to the ratio of the diffusion coefficients *D* for CO_2_ and O_2_ in water. As *k*_L_*a*_O2_ values are relatively easy to measure, the approach offers a straightforward access to *k*_L_*a*_CO2_. However, CO_2_ transfer differs fundamentally from O_2_ transport because dissociation characteristics have been taken into account (see Figure 2).

CO_2_ dissociates in water depending on pH as follows:

(5) $${\text{CO}}_{2}+{\text{H}}_{\text{2}}\text{O}\overset{k_{1}}{\underset{\;}{\underset{k_{-1}}{↔}}}{\text{H}}_{\text{2}}{\text{CO}}_{\text{3}}\overset{\text{fast}}{↔}{\text{HCO}}_{\text{3}}^{\text{-}}+{\text{H}}^{\text{+}}\overset{\text{fast}}{↔}{\text{CO}}_{\text{3}}^{\text{2-}}\text{+}2{\text{H}}^{+}$$

Because the equilibrium of CO_2_ dissociation is far on the side of the anhydride (99.8%), concentrations of the carbonic acid H_2_CO_3_ are low not exceeding one digit micromolar ranges at typical cultivation conditions. Consequently, the apparent equilibrium constant *K*_1_ (Bailey and Ollis, 1986):

(6) $$K_{1}=\frac{\left[H^{\text{+}}\right]\left[{\text{HCO}}_{\text{3}}^{\text{-}}\right]}{\left[{\text{CO}}_{\text{2}}\right]\text{+}\left[H_{\text{2}}{\text{CO}}_{\text{3}}\right]}≅\frac{\left[H^{\text{+}}\right]\left[{\text{HCO}}_{\text{3}}^{\text{-}}\right]}{\left[{\text{CO}}_{\text{2}}\right]}=10^{-6.3}M$$

is formulated and completed by Bailey and Ollis (1986) as follows:

(7) $$K_{2}=\frac{\left[{\text{H}}^{\text{+}}\right]\left[{\text{CO}}_{\text{3}}^{\text{2-}}\right]}{\left[{\text{HCO}}_{\text{3}}^{\text{-}}\right]}=10^{-10.25}\text{M}$$

One may safely assume that (de-) protonating reactions of formula (5) are very fast. However, formation and dissociation of carbonic acid from CO_2_ are suspected to limit the total equilibration process. *k*_1_ and *k*_−1_ were estimated as 0.03 1/s and 20 1/s, respectively (Bailey and Ollis, 1986).

At typical cultivation conditions (pH 7), 83.3% of the CO_2_ species are present as ${\text{HCO}}_{3}^{-},$ only 16.7% as CO_2_. Hence ${\text{HCO}}_{3}^{-}$ is about five-fold higher concentrated than CO_2_. This statement not only holds for the cultivation medium, but it should also be valid for intracellular conditions because cells aim at maintaining their intracellular pH at about this level.

**Figure 3:**
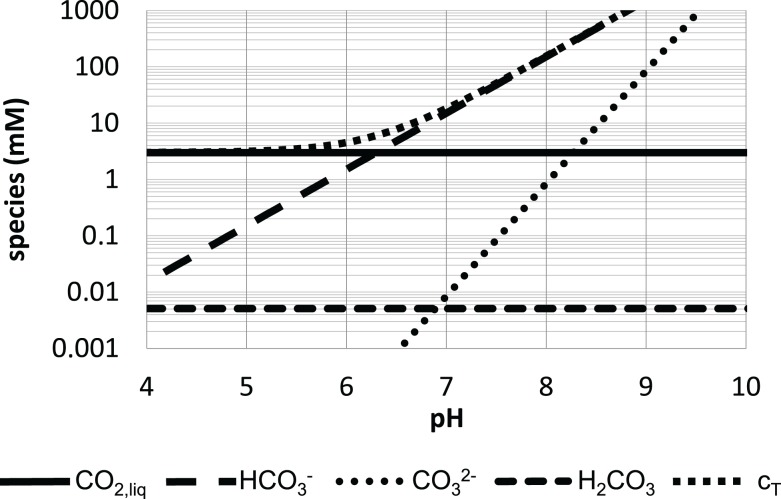
**Distribution of CO_2_ species in water at atmospheric conditions (25°C)**. The species according to formula (5) are given as well as their sum *c*_T_ [figure modified after Bailey and Ollis (1986)].

Figure 3 underpins that the full consideration of the individual species CO_2_, ${\text{HCO}}_{3}^{-}$, and ${\text{CO}}_{\text{3}}^{\text{2}-}$is crucial to get accurate values for total CO_2_ c_T_ dissolved in the fermentation suspension. Recently, Buchholz et al. (2014a) outlined that ignoring the anions leads to a carbon gap of about 20% during the first hours of fermentation. Noteworthy, the dissolved CO_2_ level is not dependent on pH (see Figure 3). According to Henry’s law only partial pressure (and salt conditions) may effect *c*_CO2_. Hence, large-scale bioreactors which have high hydrostatic pressures of 1–1.5 bar possess higher dissolved CO_2_ levels than comparable laboratory systems. This not only induces regulatory responses in the cells but also affects the buffering capacity of the large-scale suspension. Due to increased ${\text{CO}}_{\text{2}}/{\text{HCO}}_{\text{3}}^{-}$ levels, pH buffering is severely increased in large scale compared to lab fermentations.

## Metabolic Release and Incorporation

**Figure 4:**
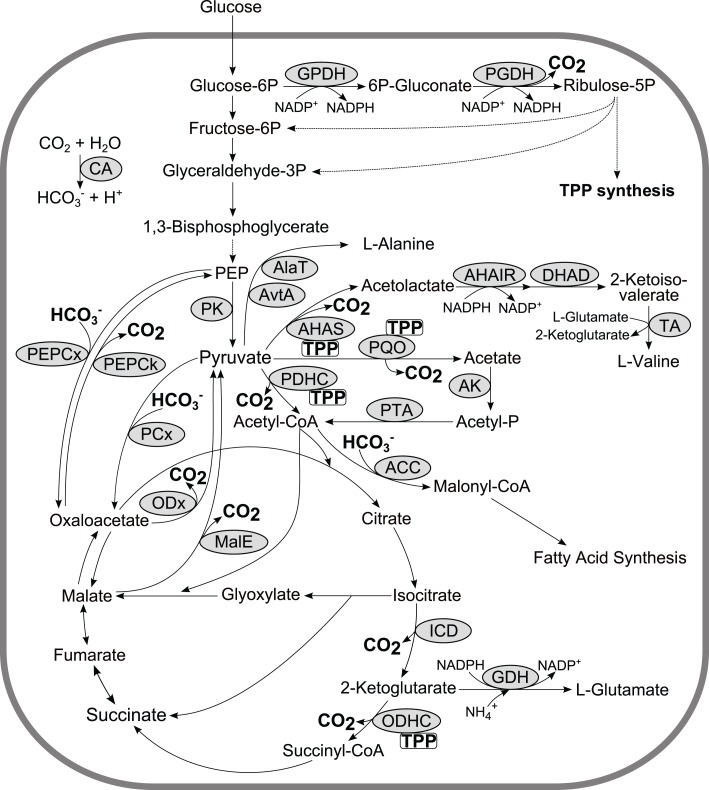
**Exemplary, a schematic overview of the central metabolism of *C. glutamicum* including carboxylases and TPP-dependant decarboxylases**. Abbreviations: ACC, acetyl-CoA carboxylase; AHAIR, acetohydroxyacid isomeroreductase; AHAS, acetohydroxyacid synthase; AK, acetate kinase; AlaT, alanine aminotransferase; AvtA, valine-pyruvate aminotransferase; CA, carbonic anhydrase; DHAD, dihydroxyacid dehydratase; GDH, glutamate dehydrogenase; GPDH, glucose-6P dehydrogenase; ICD, isocitrate dehydrogenase; MalE, malic enzyme; ODHC, 2-ketoglutarate dehydrogenase complex; ODx, oxaloacetate decarboxylase; PCx, pyruvate carboxylase; PDHC, pyruvate dehydrogenase complex; PEP phosphoenolpyruvate; PEPCk, PEP carboxykinase; PEPCx, PEP carboxylase; PGDH, 6P-gluconate dehydrogenase; PK, pyruvate kinase; PQO, pyruvate:quinone oxidoreductase; PTA, phosphotransacetylase; TA, transaminase B; TPP, thiamine pyrophosphate.

Metabolism of all living organisms is equipped with a set of carboxylases incorporating CO_2_ or bicarbonate ${\text{(HCO}}_{3}^{-})$ into organic molecules and decarboxylases releasing CO_2_ in the environment. Consequently, these fundamental reactions are directly involved in and/or interconnect anabolism, catabolism, and energy metabolism of the cell. Especially, the phosphoenolpyruvate–pyruvate–oxaloacetate node comprises an organism-specific configuration of carboxylating (e.g., pyruvate carboxylase (PCx), PEP carboxylase, acetyl-CoA carboxylase) and decarboxylating (e.g., PEP carboxykinase; malic enzyme, oxaloacetate decarboxylase; pyruvate dehydrogenase complex, pyruvate:quinone oxidoreductase) reactions (Figure 4) which are of major importance for the carbon flux distribution in the central metabolism. For instance during sugar catabolism anaplerotic C3 (phosphoenolpyruvate (PEP)/pyruvate), carboxylation and decarboxylation of pyruvate to acetyl-CoA are essentially required to maintain TCA flux whereas gluconeogenesis relies on C4 (oxaloacetate/malate) decarboxylation (Sauer and Eikmanns, 2005). Another example is pyruvate decarboxylase of yeast which is the key enzyme in ethanol fermentation and is essentially required to maintain a balanced metabolism in mineral media containing glucose as sole carbon source (Pronk et al., 1996).

Carboxylases catalyzing the thermodynamically expensive assimilation of ${\text{CO}}_{\text{2}}/{\text{HCO}}_{\text{3}}^{-}$ have been classified regarding their physiological function into autotrophic, assimilatory, biosynthetic, anaplerotic, and redox balancing enzymes (Erb, 2011). Currently, six pathways for CO_2_ fixation have been identified: the reductive pentose phosphate (Calvin–Benson) cycle, the reductive acetyl-CoA (Wood–Ljungdahl) pathway, the reductive citric acid cycle, the 3-hydroxypropionate bicycle, the dicarboxylate/4-­hydroxybutyrate cycle, and the 3-hydroxypropionate/4-­hydroxybutyrate cycle (Erb, 2011; Fuchs, 2011). Additionally, to increase the carbon fixation rate, novel synthetic pathways have been proposed (Bar-Even et al., 2010). Plants, algae and phototrophic prokaryotes possess ribulose-1,5-bisphosphate carboxylase/oxygenase (RubisCO) quantitatively the most abundant enzyme in the biosphere and the key enzyme in autotrophic CO_2_ fixation by the Calvin–Benson cycle (Miziorko and Lorimer, 1983; Hartman and Harpel, 1994; Erb, 2011). Notably, the Wood–Ljungdahl pathway is the only one which fixes CO_2_ and simultaneously generates ATP by conversion of acetyl-CoA to acetate (Fuchs, 2011) rendering this route attractive and promising for CO_2_-based microbial production purposes (Dürre and Eikmanns, 2015).

Owing to the metabolic key functions carboxylases and decarboxylases fulfill, relevant microbial, mammalian, and plant enzymes have been biochemically characterized and their regulation analyzed [e.g., Miziorko and Lorimer (1983), Chollet et al. (1996), Hanson and Reshef (1997), Nikolau et al. (2003), Sauer and Eikmanns (2005), and Jitrapakdee et al. (2006)]. ${\text{CO}}_{\text{2}}/{\text{HCO}}_{\text{3}}^{-}$-related allosteric regulation and biochemical properties of relevant enzymes have been analyzed to some extent (Jones and Greenfield, 1982), however, our understanding is limited by far, yet. The industrially important Gram-positive *C. glutamicum* possesses the biotin-dependent PCx and the PEP carboxylase, both with Michaelis–Menten constants (*K*_M_) for ${\text{HCO}}_{3}^{-}$ of about 3 mM (Hanke et al., 2005; Chen et al., 2013) which is about 30-fold higher compared to the *K*_M_ (0.1 mM i.e., 4.4 mg/L) of PEP carboxylase from *Escherichia coli* as single anaplerotic enzyme (Kai et al., 1999). These differences already point to organism-specific aeration needs to install proper metabolic activity by maintaining sufficient ${\text{CO}}_{\text{2}}/{\text{HCO}}_{\text{3}}^{-}$ availability at fermentation start when biomass concentrations are still low (Repaske et al., 1974; Talley and Baugh, 1975) or for products requiring a high anaplerotic flux (e.g., succinate, l-lysine, and derived products).

Due to the diffusive potential of CO_2_ and the rather slow chemical conversion of CO_2_ to ${\text{HCO}}_{3}^{-}$ (Kern, 1960), nature has independently evolved three classes (designated as α, β, and γ) of zinc-dependant carbonic anhydrases (CAs) which catalyze with very high turnover numbers (up to 10^6^ s^−1^) the reversible hydration of CO_2_ (Figure 4; Tashian, 1989; Tripp et al., 2001). CAs are widespread over all kingdoms of life and play a vital role in various cellular functions such as photosynthesis, ion transport, and pH homeostasis (Smith and Ferry, 2000; Merlin et al., 2003; Mitsuhashi et al., 2004). Essentially, CAs maintain adequate ${\text{HCO}}_{3}^{-}$ levels for aerobic growth under ordinary atmospheric conditions, since inactivation of CAs in several organisms such as *C. glutamicum, E. coli, Ralstonia eutropha, Candida albicans, Saccharomyces cerevisiae*, and *Aspergillus nidulans* is lethal unless the CO_2_ content in the atmosphere is significantly increased (about 5–10%; Mitsuhashi et al., 2004; Merlin et al., 2003; Kusian et al., 2002; Götz et al., 1999; Cottier et al., 2012).

In mammals, mitochondrial respiration generates CO_2_ as waste product which has to be actively transported from tissue to the depolluting lungs by the blood. Since ${\text{HCO}}_{3}^{-}$ is in contrast to CO_2_ not permeable, mammalian cells are equipped with about 13 genes encoding different types of bicarbonate transporters allowing the intercellular exchange of the former species (Casey, 2006). By contrast, ${\text{HCO}}_{3}^{-}$ transport in prokaryotes has been rarely observed so far with the well-studied exception of the bicarbonate transport system of the cyanobacteria *Synechococcus sp*. strain PCC7942. Two different transport mechanisms for ${\text{HCO}}_{3}^{-}$ maintain with the combined action of CA elevated levels of CO_2_ in the carboxysomes required for efficient carbon fixation by RubisCO (Ritchie et al., 1996; Badger and Price, 2003).

## CO_2_ Induced Growth Phenotypes

CO_2_ is the final respiratory product and consequently inevitable in aerobic microbial and mammalian bioprocesses. In exhaust gas flows the CO_2_ fraction may rise to 15–20% depending on aeration and cellular activity. Considering that head overpressures of microbial fermentations are commonly 0.5–2 bar and 10–15 m bioreactor filling heights create hydrostatic pressures of 1–1.5 bar, p_CO2_ could achieve maximum values of 0.1–0.6 bar at the bottom of the bioreactor. Notably, these maximum values may be reduced if aeration with fresh air is properly installed there. In principle, the scenario is similar for mammalian cultures although lowered due to reduced cell activities and smaller bioreactor sizes compared to microbial applications (p_CO2_ at mammalian production: about 0.180 bar_CO2_; Zhu et al., 2005). Noteworthy, cells circulating in large-scale bioreactors experience frequently changing p_CO2_ levels, a fact that is usually not simulated by pseudo-stationary scale-down tests.

Multiple studies have been performed for elucidating the impact of p_CO2_ levels on microbial (Dixon and Kell, 1989) and mammalian performance [e.g., Gray et al. (1996)]. Effects on growth, biomass per substrate yields, product formation, cell division, and morphology were analyzed. These were either attributed to elevated CO_2_ partial pressures alone or in conjunction with co-effects such as changing osmolality in the media. Observed phenotypes are individual. Nevertheless, some characteristic examples are given in the following highlighting basic kinetics of industrially interesting strains:

### Bacteria

First indications that bacteria do react on elevated dissolved CO_2_ levels were published by Jones and Greenfield (1982). Among others, they observed that growth of *Bacillus subtilis* was inhibited by 40% under p_CO2_ = 0.17 atm (0.172 bar). Batch studies with *E. coli* using CO_2_-enriched aeration revealed that the maximum growth rate was severely reduced and biomass per glucose yields increased for aeration fractions >20% of carbon dioxide (Castan et al., 2002). Baez et al. (2009) studied GFP producing *E. coli* at constant p_CO2_ in the range of 20–300 mbar. Their results supported previous findings by measuring more than 30% reduction of the maximal growth rate μ_max_ and doubled acetate formation under p_CO2_ = 300 mbar compared to the reference. For *C. glutamicum*, Knoll et al. (2005) investigated the growth rates μ in overpressurized bioreactors (10 bar head pressure) during growth on glucose. They observed μ >0.3 1/h under p_CO2_ = 0.43 bar. This finding was supported by subsequent studies with an l-lysine producing *C. glutamicum* strain (Knoll et al., 2007). Additionally, turbidostatic continuous cultivations were performed installing different p_CO2_ levels. The growth rate of 0.58 1/h turned out to be almost constant until 0.18 bar p_CO2_ and steadily decreased to 0.36 1/h under 0.8 bar_CO2_ (Bäumchen et al., 2007). In 2013, Blombach et al. studied the growth performance of *C. glutamicum* in batch cultures. While no significant growth phenotype was found installing pCO_2_ of about 0.3 bar, low levels smaller than 50 mbar_CO2_ revealed 3-phase, bi-level growth kinetics of *C. glutamicum* (Blombach et al., 2013). Recently, Lopes et al. (2014) reviewed some microbial phenotypes as a result of elevated carbon dioxide levels in over-pressurized bioreactors.

### Yeast

Chen and Gutmains (1976) reported about growth inhibition of yeast at high CO_2_ partial pressures. They found “slight” growth inhibition using CO_2_ aeration fractions of 40% and a severe growth decrease using 50% CO_2_ enriched air. Later, Kuriyama et al. (1993) underlined these early findings by arguing that cell division of *S. cerevisiae* may be hampered under p_CO2_ = 0.5 atm (0.51 bar). Kuriyama et al. (1993) used chemostat approaches for studying the p_CO2_ impact. They found that an elevated p_CO2_ coincided with increased ethanol formation which itself may hamper process performance. *S. cerevisiae* is able to adapt to hyperbaric conditions (10 bar) provided that sufficient time for adaptations is given (Belo et al., 2003). CO_2_ partial pressures of 0.48 bar had negligible effects on cell viability. This was also observed by Knoll et al. (2007). However, if partial pressures are increased further (0.6 bar) cell budding is hampered (Coelho et al., 2004). Indeed, a growth reduction of 25% was reported by Aguilera et al. (2005) when the CO_2_ fraction of aeration was increased to 79% in aerobic cultivations. However, growth under anaerobic conditions was much less affected indicating that the respiratory metabolism is likely to be more influenced under high pCO_2_ levels. This phenomenon was in the focus of recent studies. Richard et al. (2014) outlined that transient metabolic responses are triggered by CO_2_ shifts e.g., characterized by intermediary increase of respiration rates and the excretion of ethanol and acetate.

### Fungi

Similar to bacteria and yeast, inhibition of growth (and product formation) was also observed for fungi such as *Penicillium chrysogenum* already under p_CO2_ = 0.08 atm (Jones and Greenfield, 1982). Ho and Smith (1986) specified this early observation by identifying reduced growth and penicillin formation rates using 12.6% CO_2_ enriched air for cultivation. However, causes and consequences of high p_CO2_ levels on growth and product formation may not be clearly identifiable. They may rather be a matter of indirect effects finally resulting in morphology changes (McIntyre and McNeil, 1998). Also Gibbs et al. (2000) pinpointed to the chemical interaction of high p_CO2_ with precursors of penicillin biosynthesis finally deteriorating performance of *P. chrysogenum*. Nevertheless, under high levels of p_CO2_ (installed after using 10–15% enriched influent gas) increased climbing and severely reduced penicillin production were observed (El-Sabbagh et al., 2006), not only for *P. chrysogenum* but also for cephalosporin C producing *Acremonium chrysogenum* (El-Sabbagh et al., 2008).

### Mammalian cells (e.g., CHO)

Today, mammalian producers are typically derived from tissue cells giving Chinese hamster ovary (CHO) cells an outstanding importance for the production of therapeutic proteins (Pfizenmaier and Takors, 2015). It has been estimated that these cells experience p_CO2_ levels of 41–72 mbar under physiological conditions (Altman and Dittmer, 1971). However, industrial production environments are likely to impose much higher p_CO2_, especially when processes are in the focus of ongoing intensification (Ozturk, 1996). p_CO2_-induced stress usually coincides with the increase of osmolality due to titration for pH control. Hence, the interaction of both effects is often in the foreground of related studies. Kimura and Miller (1996) analyzed recombinant tissue-type plasminogen activator (tPA) production with CHO cells. Under maximum p_CO2_ of 333 mbar they observed 30% reduction of the growth rate which increased to 45% reduction in combination with high osmolality. Results of Gray et al. (1996) anticipated that an optimum for recombinant protein production exists at 40–100 mbar p_CO2_. Zhu et al. (2005) showed that industrial osmolality conditions (400–450 mOsm) together with typically high p_CO2_ (180–213 mar) levels caused a 20% drop of CHO cell viability. Besides, Takuma et al. (2007) outlined that industrial p_CO2_ values of 293 mbar reduced growth by 60% while cell-specific productivity of antibody IgG1 was almost unchanged. Additionally, there were indications that appropriate glucose limitation could compensate p_CO2_ triggered growth reduction at “moderate” 190 mbar_CO2_.

Among others, one reason for the deteriorating performance may be that protein glycosylation patterns reduce in the presence of elevated ${\text{HCO}}_{3}^{-}$ levels (Zanghi et al., 1999). Besides, DeZengotita et al. (2002) argued that glycolysis was inhibited in a dose-dependent manner when p_CO2_ levels were studied between 66 and 333 mbar in hybridoma cells. Therefore, p_CO2_ inhibition is not only a matter of CHO cells alone, but is observed for hybridoma and HEK293S cultures as well (Jardon and Garnier, 2003).

## ${\text{CO}}_{\text{2}}/{\text{HCO}}_{\text{3}}^{-}$-Induced Regulation

${\text{CO}}_{\text{2}}/{\text{HCO}}_{\text{3}}^{-}$ not only serves as substrate or product for enzymes, but also impacts the internal pH, the fluidity and permeability of membranes, and physicochemical properties of proteins, and is regarded as signal for virulence and toxin production in pathogens (Isenschmid et al., 1995; Stretton and Goodman, 1998; Follonier et al., 2013). Due to the multiple involvement of ${\text{CO}}_{\text{2}}/{\text{HCO}}_{\text{3}}^{-}$ in cellular metabolism, it seems evident that these species are directly or indirectly part of the regulatory machinery.

The human body underlies a complex ${\text{CO}}_{\text{2}}/{\text{HCO}}_{\text{3}}^{-}$ homeostasis with bicarbonate concentrations up to 140 mM in certain tissues (Arthurs and Sudhakar, 2005; Abuaita and Withey, 2009; Orlowski et al., 2013) representing a striking signal for pathogens invading the host. Although a direct association between CO_2_ and virulence is missing, Park et al. (2011) found that 10% CO_2_ stimulated aerobic growth of the human gastric pathogen *Helicobacter pylori*. CO_2_ deprivation led to increased intracellular ppGpp levels which might indicate an involvement of the stringent response in CO_2_-dependent regulation of *H. pylori’s* metabolism (Park et al., 2011). In *Vibrio cholerae* bicarbonate activates the regulatory protein ToxT which in turn induces virulence gene expression (Abuaita and Withey, 2009). Another, bicarbonate sensing transcriptional regulator is the AraC-like protein RegA from the mouse enteric pathogen *Citrobacter rodentium* which in the presence of bicarbonate activates transcription of a number of virulence genes and inhibits expression of several housekeeping genes (Yang et al., 2009). *C. albicans* a fungal pathogen causing life-threatening infections in immunocompromised patients senses increased ${\text{HCO}}_{3}^{-}$ levels by the soluble adenylyl cyclase (sAC) Cyr1p which produces cAMP. Then, cAMP activates protein kinase A to trigger filamentous growth which is an important feature for adhesion and invasion of the pathogen (Klengel et al., 2005; Hall et al., 2010). Furthermore, the transcription factor Rca1p of *C. albicans* was shown to control expression of CA in response to the availability of CO_2_ (Cottier et al., 2012). Both examples demonstrate the relevancy of a ${\text{CO}}_{\text{2}}/{\text{HCO}}_{\text{3}}^{-}$ signaling system for global regulation of *C. albicans’* metabolism. Regulation by bicarbonate-responsive soluble ACs seems be more widespread across multiple kingdoms since ${\text{CO}}_{\text{2}}/{\text{HCO}}_{\text{3}}^{-}$-dependent adjustment of the intracellular cAMP level, initially found in male germ cells, was also identified in mycobacteria, eubacteria, fungi, and cyanobacteria (Chen et al., 2000; Zippin et al., 2001; Bahn and Mühlschlegel, 2006).

Although in large-scale fermentations gradients of dissolved gases occur and high ${\text{CO}}_{\text{2}}/{\text{HCO}}_{\text{3}}^{-}$ concentrations depending on the process and the production host arise (Hermann, 2003; Takors, 2012), only few studies investigated the effects of altered levels of these species on metabolism and regulation of industrial relevant microbial cells systematically. The already mentioned analysis of Baez et al. (2009) studied the effect of 300 mbar partial pressure on recombinant GFP producing *E. coli* not only metabolically but also on the transcriptional level. Expression analysis of 16 selected genes revealed only slight changes in transcription. Noteworthy, as response to elevated dissolved CO_2_ the transcription of acid stress genes (*gadA, gadC*, and *adiA*) increased, indicating acidification of the internal pH by CO_2_ (Baez et al., 2009).

Recently, Follonier et al. (2013) exposed *Pseudomonas putida* KT2440 to elevated pressure (up to 7 bar) associated with increased ${\text{CO}}_{\text{2}}/{\text{HCO}}_{\text{3}}^{-}$ concentrations in the bioreactor. They investigated the global transcriptional response by DNA microarrays. Physiology of *P. putida* KT2440 was hardly affected at increased pressure, however, significant changes in gene transcription were observed: elevated ${\text{CO}}_{\text{2}}/{\text{HCO}}_{\text{3}}^{-}$ levels activated the heat-shock response and strongly affected expression of cell envelope genes pointing to an altered permeability/fluidity of the membrane (Follonier et al., 2013).

The genome-wide transcriptional response of *S. cerevisiae* to high CO_2_ concentrations was analyzed in chemostat cultures under aerobic and anaerobic conditions. Accompanied with a more pronounced sensitivity of respiratory metabolism, high CO_2_ levels in glucose-limited cultures led to 104 at least two-fold altered transcripts compared to 33 under anaerobic conditions. Interestingly, 50% of the affected transcripts under aerobic conditions encoded mitochondrial proteins such as PEP carboxykinase, PCx, and proteins involved in oxidative phosphorylation (Aguilera et al., 2005).

Recently, we investigated the effects of low (pCO_2_ < 40 mbar) and high (pCO_2_ ≥ 300 mbar) ${\text{CO}}_{\text{2}}/{\text{HCO}}_{\text{3}}^{-}$ levels on growth kinetics and the transcriptional response of *C. glutamicum* compared to standard conditions. Under high ${\text{CO}}_{\text{2}}/{\text{HCO}}_{\text{3}}^{-}$ levels growth kinetics were not affected albeit the biomass to substrate yield was increased. However, a complex transcriptional response involving 117 differentially expressed genes was observed. Among those, 60 genes were assigned to the complete DtxR/RipA regulon controlling iron homeostasis in *C. glutamicum*. The mutant *C. glutamicum* Δ*dtxR* showed significantly impaired growth under high ${\text{CO}}_{\text{2}}/{\text{HCO}}_{\text{3}}^{-}$ conditions (compared to the wildtype) but not under standard conditions. This finding underlines the relevancy of the master regulator for cell fitness under high ${\text{CO}}_{\text{2}}/{\text{HCO}}_{\text{3}}^{-}$ levels (Blombach et al., 2013). At low ${\text{CO}}_{\text{2}}/{\text{HCO}}_{\text{3}}^{-}$ levels *C. glutamicum* showed three distinct growth phases. In the mid-phase with slowest growth, *C. glutamicum* secreted l-alanine and l-valine into the medium and showed about two times higher activities of glucose-6-P dehydrogenase and 6-phosphoglconate dehydrogenase and a strong transcriptional response (>100 genes with altered expression) including increased transcription of almost all thiamine pyrophosphate (TPP) genes compared to standard conditions. We hypothesized that *C. glutamicum* counteracts the lack of ${\text{CO}}_{\text{2}}/{\text{HCO}}_{3}^{-}$ by triggering TPP biosynthesis for increasing the activities of TPP-dependent enzymes involved in CO_2_ formation (Figure 4; Blombach et al., 2013).

Industrial scale cells are exposed to various gradients such as pH, substrates, and dissolved gases. To analyze the effects of oscillating ${\text{CO}}_{\text{2}}/{\text{HCO}}_{3}^{-}$ levels on the metabolism and transcriptional response of *C. glutamicum*, a novel three-compartment cascade bioreactor system was developed. pCO_2_ gradients of 75–315 mbar at industry-relevant residence times of about 3.6 min did not significantly influence the growth kinetics but led to 66 differentially expressed genes compared to control conditions. Interestingly, the overall change in expression was directly linked to the pCO_2_ gradients and the residence time of the cells in the scale-down device (Buchholz et al., 2014b).

## ${\text{CO}}_{\text{2}}/{\text{HCO}}_{3}^{-}$ Impacts Production Processes

Production processes on glycolytic substrates rely on the anaplerotic function of PCx and/or PEP carboxylase to replenish citric acid cycle intermediates that are incorporated for anabolic demands and/or product formation. Especially, oxaloacetate-derived products such as l-lysine require a high anaplerotic flux. *C. glutamicum* is the workhorse in industrial l-lysine production and possesses PCx and PEP carboxylase. Several studies identified PCx and especially deregulated variants as most relevant to improve oxaloacetate supply since inactivation of PCx reduced and overexpression of the corresponding *pyc* gene significantly improved l-lysine formation in *C. glutamicum* (Peters-Wendisch et al., 2001; Ohnishi et al., 2002). Furthermore, inactivation of PEP carboxykinase led to an increase in l-lysine production with *C. glutamicum* (Riedel et al., 2001). Surprisingly, although great efforts have been made to tailor the biosynthetic pathway and to optimize precursor availability (Blombach and Seibold, 2010), the impact of altered ${\text{CO}}_{\text{2}}/{\text{HCO}}_{3}^{-}$ levels for aerobic l-lysine production has not been systematically investigated so far.

Apparently too low ${\text{CO}}_{\text{2}}/{\text{HCO}}_{3}^{-}$ levels may limit the *in vivo* activity of anaplerotic reactions. The combination of high aeration and low biomass concentration at the beginning of the fermentation is likely to cause retarded cell growth due to CO_2_ over-stripping. By analogy, installing non-limiting ${\text{CO}}_{\text{2}}/{\text{HCO}}_{3}^{-}$ levels is especially important for zero-growth or resting cell bioprocesses. Examples are the synthesis of organic acids such as malate, fumarate and succinate which are formed anaerobically from oxaloacetate via the reductive arm of the citric acid cycle. Under such conditions only minor amounts of ${\text{CO}}_{\text{2}}/{\text{HCO}}_{3}^{-}$ are provided by the metabolism of the cell. However, elevated productivities can be achieved by sparging with CO_2_ or adding carbonates to the medium to ensure sufficient ${\text{HCO}}_{3}^{-}$ for C3-carboxylation (Inui et al., 2004; Okino et al., 2005, 2008; Lu et al., 2009; Zelle et al., 2010; Zhang et al., 2010; Wieschalka et al., 2012). Inui et al. (2004) and Okino et al. (2005) showed that addition of NaHCO_3_ to the medium significantly improved the glucose consumption rate and the succinate production rate with resting cells of *C. glutamicum* R. Radoš et al. (2014) demonstrated that sparging an anaerobic culture of non-growing *C. glutamicum* with CO_2_ improved the succinate and acetate yield, respectively, both at the expense of lactate production. ^13^C nuclear magnetic resonance analysis of labeling patterns in the end products verified the incorporation of bicarbonate and the formation of succinate mainly via the reductive arm of the citric acid cycle (Radoš et al., 2014). For a dual-phase (aerobic growth, anaerobic production) succinate production process with a recombinant *E. coli* strain, it was also shown that increasing the CO_2_ content in the gas phase from 0 to 50% improved the biomass-specific production rate and the succinate yield significantly (Lu et al., 2009). In order to provide additional CO_2_ and reduction equivalents for anaerobic succinate production from glucose, Litsanov et al. (2012) integrated the *fdh* gene encoding a formate dehydrogenase from *Mycobacterium vaccae* into the chromosome of an engineered *C. glutamicum* strain. Supplementation of formate increased the succinate yield by 20% mainly due to increased NADH availability. However, part of the formed CO_2_ was incorporated into the product (Litsanov et al., 2012).

The shortage of oil resources and steadily rising oil prices has stimulated efforts to produce chemicals and fuels directly from CO_2_. Production of ethanol, isobutyraldehyde, and isobutanol from CO_2_ and light was achieved using engineered photosynthetic bacteria such as *Rhodobacter capsulatus* and *Synechococcus elongates* PCC7942 (Wahlund et al., 1996; Atsumi et al., 2009). Li et al. (2012) showed the feasibility of electrochemical supply of electrons to produce isobutanol and 3-methyl-1-butanol from CO_2_ with engineered *R. eutropha* H16. However, the low productivity and final titer of such approaches and the reactor design is still a challenge for future industrial application. Alternatively, RubisCO was functionally expressed in heterotrophic *S. cerevisiae* to incorporate CO_2_ as co-substrate improving ethanol production and reducing the formation of the by-product glycerol in chemostat cultures (Guadalupe-Medina et al., 2013). An innovative approach is the use of CO_2_ and hydrogen-containing waste gases or synthesis gas as feedstock for the production of chemicals and fuels with acetogenic and carboxydotrophic bacteria. Aerobic and anaerobic gas fermentation processes have been exploited for their biotechnological potential and commercial plants for ethanol production are already under construction (Dürre and Eikmanns, 2015).

Mammalian producer cells descent from rodents (like mouse or hamster) or human tissues. In case they are used in submerse culture they have undergone a (sometimes) tedious transition to yield at suspended producer cell lines. With this history in mind one may understand why product formation in producer cells such as CHO is often found to be strongly growth de-coupled (Altamirano et al., 2001). This fact is even exploited by temperature shift-down approaches (37 to ~30°C) to arrest cells in G1 phase finally increasing cell-specific protein production. By analogy, osmolality increase results at similar growth and product formation phenotypes (Ozturk and Palsson, 1991; Kumar et al., 2007). As outlined in the foregoing sections, elevated pCO_2_ environments >100 mbar are likely to inhibit cell growth for CHO cultures. Consequently, therapeutic protein formation kinetics of the (typical) growth de-coupled type are not likely to be affected by high pCO_2_ environments. Indeed, findings of Takuma et al. (2007) support this conclusion. In case growth-coupled product formation is observed, the impact of increased carbon dioxide partial pressures may be more pronounced. This holds also true for putative interactions of high ${\text{CO}}_{\text{2}}/{\text{HCO}}_{\text{3}}^{-}$ levels with the cellular membrane or the product proteins. However, more studies are necessary to investigate these individual effects.

## Conclusion

Summarizing the impacts of high ${\text{CO}}_{\text{2}}/{\text{HCO}}_{\text{3}}^{-}$ levels, the reduction of cellular growth is a typical phenomenon. Although the effects are very individual, sensitivities on high CO_2_ partial pressures are less pronounced in bacteria than they are in fungi or mammalian producer cells. As a rule of thumb pCO_2_ > 100 mbar marks the beginning of growth inhibition for the later.

On the other hand, too low ${\text{CO}}_{\text{2}}/{\text{HCO}}_{\text{3}}^{-}$ levels are likely to limit anaplerotic reactions inside the cells. Consequently, downstream precursors such as oxaloacetate could become limiting which affects not only cell growth but also biosynthesis of related metabolic products.

In general, transcriptional responses on high (or low) ${\text{CO}}_{\text{2}}/{\text{HCO}}_{\text{3}}^{-}$ are by far less studied than metabolic phenotypes. However, (maybe) surprising regulatory mechanisms are waiting to be discovered. An illustrative example is the case of *C. glutamicum* that aims at counteracting ${\text{CO}}_{\text{2}}/{\text{HCO}}_{\text{3}}^{-}$ limitation by amplifying TPP biosynthesis, known as an essential co-factor for decarboxylating enzymes. High ${\text{CO}}_{\text{2}}/{\text{HCO}}_{\text{3}}^{-}$ levels apparently serve as an important stimulus for some pathogenic microbes to identify the host and to trigger related invasion programs. To what extent fragments or derivatives of such regulatory scenarios are also present in other cells also remains to be discovered.

Considering the application of microbes, yeasts, fungi, and mammalian cells in industrial bioreactors some particularities need to be taken into account. High ${\text{CO}}_{\text{2}}/{\text{HCO}}_{\text{3}}^{-}$ levels do not effect cells as a singular, isolated event. They rather occur in conjunction with changes of osmolality and pH that stimulate the cells manifold. The unequivocal identification of causes and consequences may be hampered intrinsically. Complex networking analysis is necessary to decipher details of ${\text{CO}}_{\text{2}}/{\text{HCO}}_{\text{3}}^{-}$ impacts. Examples are the link between ${\text{CO}}_{\text{2}}/{\text{HCO}}_{\text{3}}^{-}$ and productivity with morphology changes in fungi or the osmolality in CHO. On the other side, equal pCO_2_ levels may serve as a valuable scale-up criterion because they mirror the complex interaction of cellular activities, mixing, and mass transfer (Klinger et al., 2015). Furthermore, one should consider that ${\text{CO}}_{\text{2}}/{\text{HCO}}_{\text{3}}^{-}$ stimuli occur dynamically under industrial operation conditions. Cells are circulating in large-scale production reactors thus experiencing frequently changing dissolved CO_2_ levels. Consequently, comprehensive scale-up tests should mirror these conditions to ensure that promising novel producers will perform equally well in large scale – as they should.

## Conflict of Interest Statement

The authors declare that the research was conducted in the absence of any commercial or financial relationships that could be construed as a potential conflict of interest.
